# Fetal Cardiac Hemodynamic and Sonographic Anomalies in Maternal COVID-19 Infection Depending on Vaccination Status—Polish Multicenter Cohort Study

**DOI:** 10.3390/jcm12165186

**Published:** 2023-08-09

**Authors:** Iwona Strzelecka, Oskar Sylwestrzak, Julia Murlewska, Jerzy Węgrzynowski, Katarzyna Leszczyńska, Krzysztof Preis, Maria Respondek-Liberska

**Affiliations:** 1 Department of Fetal Malformations Diagnosis and Prevention, Medical University of Łódź, 90-419 Lodz, Poland; 2 Department of Prenatal Cardiology, Polish Mother’s Memorial Hospital Research Institute in Łódź, 93-338 Lodz, Poland; 3 Private Gynecology and Obstetrics Clinic, 60-502 Poznań, Poland; 4 Department of Obstetrics and Gynecology, Zdroje Hospital, 70-780 Szczecin, Poland; 5 Department of Obstetrics, Medical University of Gdansk, 80-210 Gdansk, Poland; 6 Department of Obstetrics, Gynecological Diseases and Oncological Gynecology of the Regional Hospital in Toruń, 87-100 Toruń, Poland

**Keywords:** SARS-CoV-2, COVID, fetal ultrasonography, fetal echocardiography, prenatal cardiology

## Abstract

Most obstetrical studies have focused on maternal response to the SARS-CoV-2 virus but much less is known about the effect of COVID-19 on fetal physiology. We aimed to evaluate the effect of the maternal SARS-CoV-2 infection on the fetal homeostasis with the use of detailed ultrasonography and echocardiography and consideration of the effect of vaccination. This was a multi-center study of fetuses who had prenatal detailed ultrasound and echocardiographic examinations performed by fetal cardiology specialists. The subjects were divided based on the COVID vaccination status (vaccinated women who did not have COVID-group V, unvaccinated women who had COVID-group UV, and unvaccinated women who did not have COVID-control group). We evaluated the ultrasound and echocardiography results obtained. The study group included 237 gravidas from four prenatal cardiology centers. In the group of fetuses with normal heart anatomy, normal cardiovascular function had 147 (81%) fetuses and functional cardiovascular anomalies were present in 35 (19%) cases. Functional cardiovascular anomalies were present in 11 (16%) fetuses in the V group, 19 (47%) fetuses in the UV group and 5 (8%) fetuses in the control group (*p* < 0.01). There were 56 (24%) fetuses with extracardiac anomalies. Extracardiac anomalies were present in 20 (22%) fetuses in the V group, 22 (45%) fetuses of the UV group and in 14 (14%) fetuses in the control group (*p* < 0.01). Our study has proved that maternal COVID-19 infection can affect the fetal physiology and mild cardiac and extracardiac markers detected by fetal ultrasonography and echocardiography. Moreover, maternal vaccination results in lower occurrence of these findings in fetuses.

## 1. Introduction

The SARS-CoV-2 coronavirus belongs to the group of coronaviruses (coronaviridae). This is a group of viruses that by the end of the twentieth century caused infections mainly in animals and a few species also in humans. They involved mainly mild infections of the upper respiratory tract and sometimes the gastrointestinal tract. So far, about 15–30% of mild seasonal “colds” have been caused by coronaviruses. At the turn of 2002 and 2003, a new subspecies of coronavirus appeared in China and some people were affected by the symptoms of acute respiratory failure—this virus was called SARS-CoV (severe acute respiratory syndrome). The restrictions related to the SARS-CoV-2 pandemic and the COVID-19 disease are slowly disappearing, but it seems that the virus has not yet said the last word and its next mutations will stay with us forever, like the flu virus. The course of infections caused by subsequent mutations will be less severe and more dangerous only for immunodeficient people, the elderly and pregnant women. Vaccination will protect against severe infection. Vaccination recommendations against the influenza virus has already been included in the pregnancy schedule. We would like such recommendations also to be adopted in relation to vaccination against COVID-19. In December 2019, a novel corona virus called SARS-CoV-2 emerged in China and caused significant public health and economic problems. COVID-19 still remains a challenging problem for clinicians because of its acute and chronic effects [1,2,3]. Fortunately, the vaccines were found to be a great breakthrough by preventing SARS-CoV-2 infection in vaccinated individuals, reducing disease severity and, ultimately, preventing death in vaccinated individuals [4,5]. Pregnancy implicates an immunosuppressive state and physiological changes (diaphragm elevation, increased oxygen consumption, and edema of respiratory tract mucosa), which lead to susceptibility to pathogens and severe pneumonia. That is why pregnant women may have been more prone to severe infection [6,7,8]. So far, most of the obstetrical studies have focused on maternal response to the SARS-CoV-2 virus [9,10,11,12]; however, much less is known about the effect of COVID-19 on fetal physiology. In this study, we aimed to evaluate the effects of the maternal SARS-CoV-2 infection on the fetal homeostasis with the use of detailed ultrasonography and echocardiography and consideration of the effect of maternal vaccination.

## 2. Material and Methods

This was a multi-center study of fetuses who had detailed prenatal ultrasound and echocardiographic examinations performed by fetal cardiology specialists (certified by the Polish Prenatal Cardiology Society). The study was conducted in four Polish centers (Łódź, Szczecin, Gdańsk, Poznań) from October 2021 to May 2022. These centers sent a protocol from examinations to the main center. The Polish centers of prenatal cardiology have rank C or B in the prenatal cardiology system in Poland (www.orpkp.pl) (accessed on 20 October 2022), which means that they are referral centers (mostly patients with high-risk pregnancies; more than 100 congenital heart defect cases per year) and perform referral fetal echocardiography and”genetic” ultrasonography examinations [13]. The data of the patients were de-identified and this was an observational study and we did not change the method of medical management but focused on the interpretation of additional collected data, thus; a new approval from the Ethical Committee was not necessary. The subjects were divided based on their COVID vaccination status (vaccinated women who did not have COVID-group V, unvaccinated women who had COVID-group UV, and unvaccinated women who did not have COVID-control group). The study group included 237 gravidas from four prenatal cardiology centers. There were 88 vaccinated gravidas who did not have COVID-19 (group V), 49 unvaccinated gravidas who had COVID-19 (group UV) and 100 unvaccinated gravidas (control group) who did not have COVID-19. The mean age of the gravidas was 31 ± 5 years (group V 32 ± 5 years [range of age: 21–45); group UV 32 ± 5 years [range of age: 22–44]; control group 30 ± 5 years [range of age: 19–44). The appropriate figures and tables were prepared in Word and Microsoft Excel 365 software. The clinical status of pregnant women with SARS-CoV-2 infection was moderate. Some patients were vaccinated before pregnancy and some during pregnancy. For echocardiographic screening, relevant built-in echocardiographic programs in the ultrasound machines were used. For the assessment of the flow of blood, color and spectral Doppler methods were applied.

Exclusion criteria included: (1) no fetal echocardiography; (2) improper technique of echocardiography or poor quality of examination; (3) lack of vital data. We excluded cases with maternal urogenital infections. Cardiac functional abnormalities included tricuspid insufficiency, pericardial effusion, myocardial hypertrophy, bright spot, mild cardiomegaly, abnormal foramen ovale blood flow, reversal flow in the ductus arteriosus or mild arrhythmia. Extracardiac anomalies included a thick, edematous placenta, an umbilical cord around the fetal neck, oligohydramnios, polihydramnios, pyelectasis, hiperechogenic bowels, a renal cyst, a lung cyst, hiperechogenic lungs, bowels distension, hydrops testis, mild ventriculomegaly, a visible bronchogram, a vacuolised or thick placenta > 5 cm.

## 3. Results

The study group included 237 gravidas from four prenatal cardiology centers. There were 88 vaccinated gravidas who did not have COVID-19 (group V), 49 unvaccinated gravidas who had COVID-19 (group UV) and 100 unvaccinated gravidas (control group) who did not have COVID-19. The mean age of the gravidas was 31 ± 5 years (group V 32 ± 5 years [range of age: 21–45]; group UV 32 ± 5 years [range of age: 22–44]; control group 30 ± 5 years [range of age: 19–44]. Previous maternal obstetrical history is presented in Table 1. The gravidas in the V group were fully vaccinated with the Comirnaty (by BioNTech and Pfizer) vaccine (69 cases), Vaxzevria (by AstraZeneca AB) vaccine (9 cases), Spikevax (by Moderna Biotech Spain SL) vaccine (6 cases) and COVID-19 Vaccine Janssen (by Janssen-Cilag International NV) vaccine (4 cases). Three of the patients had obesity (with the BMI > 30). None of the women was smoking. Three women had chronic diseases. In two cases, the women presented hypothyroidism and diabetes and in one case the woman presented gestational diabetes mellitus. None of the patients had pregnancy complications and none took any medications. The echocardiographic examinations were performed at 28 ± 6 weeks of gestation (mean) according to the last menstrual period (group V 27 ± 5 weeks of gestation; group UV 31 ± 5 weeks of gestation; control group 28 ± 6 weeks of gestation). There were nine large-for-gestational-age fetuses, five small-for-gestational-age fetuses and two cases of intrauterine growth restriction.

**Figure 1:**
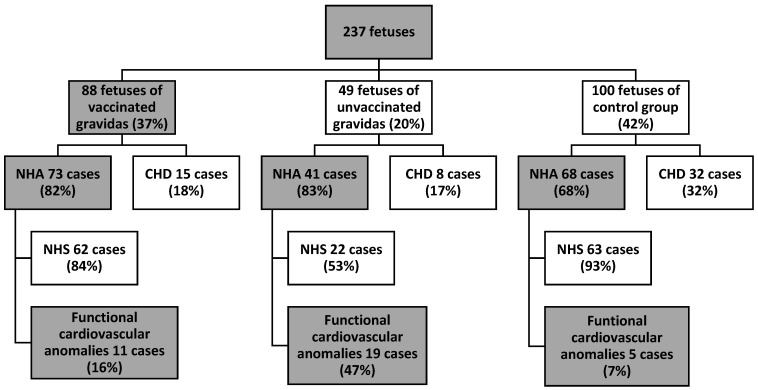
Study group presentation—fetal cardiovascular anomalies.

**Figure 2:**
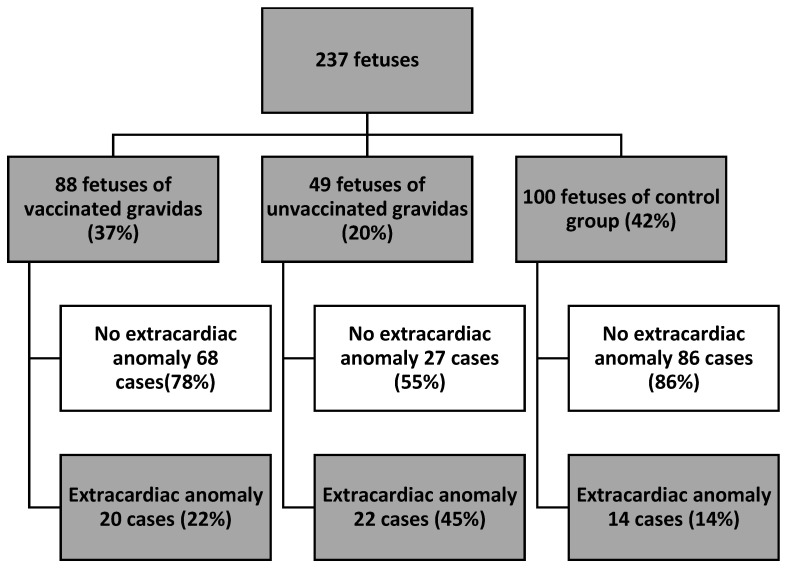
Study group presentation—fetal extracardiac anomalies (ECA—defined as anomalies that do not require surgical intervention after birth).

There were 182 (76%) fetuses with a normal heart anatomy and 55 (23%) fetuses with a congenital heart defect (Figure 1). In the group of fetuses with a normal heart anatomy, normal cardiovascular function had 147 (81%) fetuses and functional cardiovascular anomalies were present in 35 (19%) cases. Functional cardiovascular anomalies were present in 11 (16%) fetuses of group V, 19 (47%) fetuses of group UV and 5 (8%) fetuses of the control group (*p* < 0.01) (Figure 1). More detailed data are presented in Table 2. There were 56 (24%) fetuses with extracardiac anomalies. Extracardiac anomalies were present in 20 (22%) fetuses in the V group, 22 (45%) fetuses in the UV group and in 14 (14%) fetuses in the control group (*p* < 0.01) (Figure 2). More detailed data are presented in Table 3.

## 4. Discussion

Maternal COVID-19 infection can affect the fetal physiology and mild cardiac and extracardiac markers detected by fetal ultrasonography and echocardiography. Maternal vaccination results in lower occurrence of these findings in fetuses. Diverse viruses are known to cause fetal infections. Rubella, cytomegalovirus (CMV), herpes simplex virus (HSV), and human immunodeficiency virus (HIV) could enter the amniotic cavity even if amniotic membranes are intact. Others could enter the fetus via the hematogenous route. Herpes simplex virus (HSV), varicella zoster virus (VZV), and Coxsackievirus are among those viruses which can cross the placental barrier and infect the fetus [14]. Viral infections may cause fetal malformations and affect, for example, the fetal central nervous system and present a wide range of brain pathologies or may remain asymptomatic during the fetal life and be undetected by ultrasonography [15,16]. The signs of fetal infection may be subtle and present as intrauterine growth restriction, calcifications of fetal organs, placenta, cord, and membranes and failure to adequately develop fetal fat reserves [14,17,18]. Even though diagnosis of the fetal infection must be confirmed by identification of a pathogen by means of microbiological cultures, immunological techniques and special molecular biology techniques, fetal ultrasonography and echocardiography in these cases seem reasonable to monitor the fetal well-being and assess the fetal cardiovascular efficacy [19,20]. So far, SARS-CoV-2 infection has affected pregnant women and the course of pregnancy. Although the course of COVID-19 was mild in the majority of cases, it was also associated with a 0.8% rate of maternal mortality and 11.1% rate of admission to the intensive care unit. A total of 4.1% of the patients had preterm delivery, whereas miscarriage was observed in 2.2% of patients, which was due to the most common pregnancy complications. All neonates were negative for COVID-19 [21,22]. Recently, post-COVID-19 fetal cardiac evaluation was published by Goncu et al. [23]. They analyzed different fetal cardiac parameters and concluded that the fetuses of mothers who recovered from the COVID-19 infection had a similar cardiac morphology and function and only mitral E/A ratio results were found to be statistically significantly lower in this group compared to the control group. The fetal heart did not seem to be negatively affected by COVID-19 after recovery from a moderate infection [23]. Another Turkish study showed that the fetal cardiac output was significantly reduced in pregnant women who recovered from SARS-CoV-2 infection and had a severe course of the disease, while there was no significant difference in mild and moderate cases [24]. Additionally, earlier gestational age at birth, lower birth weight, preterm births and fetal distress symptoms were observed in a group of patients with severe infection; however, this study included a small study group [24]. As in the previous studies, we found that SARS-CoV-2 may be related to fetal physiology alterations due to the fact that more cardiac and extracardiac anomalies were found in the UV group. Now, our study has focused on a more descriptive analysis, not a quantitative one. Interestingly, a study published in the *Prenatal Cardiology Journal* hypothesizes that an increase in fetal-isolated pericardial effusion coincided with the period of massive COVID-19 immunization, as a result of a transient fetal inflammatory response to SARS-CoV-2 immunization [25].

**Figure 3:**
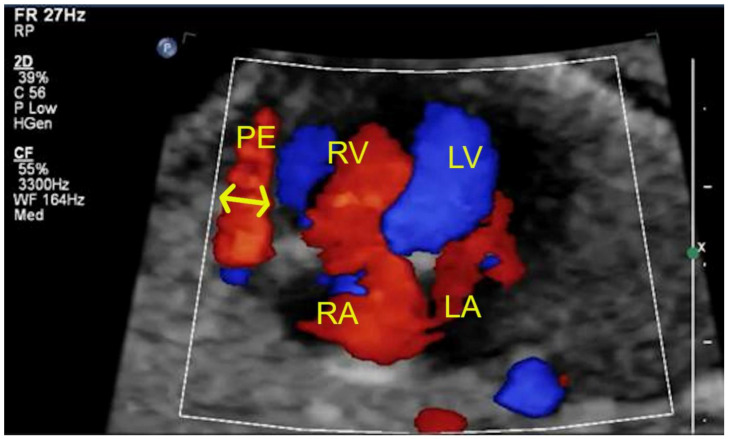
Fetal pericardial effusion during maternal COVID-19 virus infection.

Our results showed that in the V group (vaccinated), there was only one case of fetal pericardial effusion (Figure 3) but in the UV group (unvaccinated) there were more cases of fetal pericardial effusion than in the V group. According to the authors, these findings may be complementary to one another. Perhaps the fetal pericardial effusion is related to the transient fetal immunization; however, such immunization is more prominent (a more extensive immunological process) in the case of the COVID-19 infection compared to the COVID-19 vaccination. That would also explain the lower occurrence of fetal cardiac and extracardiac anomalies as mild markers overall in the V group than in the UV group. The study also presented a high percentage of fetuses with CHD. This was caused by the specialty of our referral centers dealing with many congenital defects in everyday practice. That is why the fetuses with CHD were excluded from comparison of functional cardiac abnormalities. Describing and analyzing functional cardiac abnormalities in the case of fetal CHD is a more profound subject and could not be compared with functional cardiac abnormalities in the case of a normal heart anatomy. Extracardiac malformations such as agenesis of corpus callosum, unilateral renal agenesis, gastroschisis or myelomeningocele were also excluded from the analysis for the same reason.

In our country, the National Society of Gynecologists and Obstetricians recommends the COVID-19 vaccination [26]. COVID-19 vaccines generate robust humoral immunity in pregnant women. Immune transfer also occurs via the placental way [27]. The data of the biggest companies producing COVID-19 vaccines have found no safety concerns with no adverse effect on female reproduction, fertility, fetal or embryonal, or postnatal development, or miscarriage [28]. Nevertheless, vaccines may still not be accepted by pregnant women around the world. The strongest predictors of vaccine acceptance included confidence in vaccine safety or effectiveness, concerns about COVID-19, belief in the importance of vaccines to their own country, compliance with mask guidelines, trust in public health agencies/health science, as well as attitudes towards routine vaccines [29]. That acceptance and its predictors among women differ on a global scale [29]. Working in a healthcare system was not associated with vaccine acceptance [30]. In general, infection, which in the case of COVID-19 may be prevented by vaccination, may be connected with elevated levels of cytokines in the amniotic fluid: interleukin-1β (IL-1β), interleukin-6 (IL-6), and prostaglandins, such as PGE2 and PGE2a [31]. Microorganisms also induce the production of IL-8, CCL-2, TNF-α and some proteases [32]. Cytokines present wide pleiotropy and redundancy; their scrutinized effect on the human fetus is therefore not so easily studied and analyzed. This subject does not fall within the scope of our paper, but it seems reasonable to analyze fetal response to the altered intrauterine environment, especially when COVID-19 infection is so widespread. In our study, vaccination of the mother showed negative correlation with the occurrence of fetal cardiac and extracardiac anomalies. Even though the observed functional anomalies did not affect significantly fetal cardiovascular efficacy, their occurrence may be related to the fetal response to COVID-19 infection.

There are some important clinical implications resulting from our study. Recommendations for vaccination against the influenza virus have already been included in the schedule of vaccinations for pregnant women. This study may contribute to an elaboration of recommendations for vaccination against COVID-19. It is very important due to the fact that there have been many myths about the COVID-19 vaccination in our society. The increase in the awareness of patients about possible benefits of the COVID-19 vaccination may make patients more willing to be vaccinated and thus decrease the side-effects of any complications following COVID-19 infection.

A key strength of this paper is that is includes a large study group of carefully selected patients. This study shows a current problem that covers the psychosomatic space and the unforeseen long-term effects of COVID-19 on the health of people. The authors determine the impact of maternal SARS-CoV-2 infection on fetal homeostasis with the use of detailed ultrasound and echocardiographic examinations as well as a maternal vaccination. The awareness of the presence of the above-described ultrasound and echocardiographic markers may increase the attention of specialists and patients and prepare a plan for further diagnosis and procedures. This study demonstrates that fetal ultrasonography and echocardiography constitute excellent non-invasive tools for assessing the overall condition of the fetus in terms of any anomalies resulting from the maternal COVID-19 infection. Ultrasonographic and echocardiographic screening can be successfully used in daily practice in patients following the SARS-CoV-2 infection.

In spite of the large amount of data, there are also some limitations that need to be addressed. The most important one is the still small group of patients and the number of clinics participating in the study. Another limitation may be the awareness of people and their beliefs about the negative effects of vaccination. However, these limitations can be considered in further studies.

## 5. Conclusions

Maternal COVID-19 infection seems to affect the fetal physiology alterations and mild cardiac and extracardiac markers detected by fetal ultrasonography and echocardiography. Maternal vaccination results in lower occurrence of these findings in fetuses. Further studies are needed to establish the direct influence of the SARS-CoV-2 infection on the fetal cardiac function.

## Figures and Tables

**Table 1:** Maternal data—past conceptional history.

Which Pregnancy?	Number of Gravidas
1	93
2	82
3	32
4	17
5	7
6	2
8	2

**Table 2:** Functional cardiovascular anomalies in study groups with normal heart anatomy (no CHD).

Functional Cardiovascular Anomaly	Group V (*n* = 72)	Group UV (*n* = 42)	Control Group (*n* = 68)
Tricuspid insufficiency (in color and in spectral Doppler)	2	8	2
Pericardial effusion (>2 mm)	1	4	-
Myocardial hypertrophy (Septal thickness > 4.5 mm)	3	1	1
Bright spot	4	4	-
Mild cardiomegaly	2	-	-
Abnormal foramen ovale blood flow (bidirectional or left–right)	2	-	-
Reversal flow in ductus arteriosus	-	1	1
Arrhythmia (PAC)	-	1	1
**Total:**	**14 (18%)**	**19 (47%)**	**5 (8%)**

PAC—premature atrial contractions.

**Table 3:** Fetal extracardiac anomalies in study groups. One case could have more than one extracardiac anomaly. (ECA—defined as anomalies that do not require surgical intervention after birth).

Extracardiac Anomaly	Group V (*n* = 88)	Group UV (*n* = 49)	Control Group (*n* = 100)
Thick placenta (>50 mm) with abnormal 3D Doppler vascularity	8	4	6
Umbilical cord around the fetal neck	4	6	2
Oligohydramnios	3	4	4
Polihydramnios	3	2	4
Pyelectasis	3	5	2
Hiperechogenic bowels	3	-	3
Renal cyst	1	-	-
Lung cyst	1	-	-
Hiperechogenic lungs	1	-	-
Bowels distension	2	2	-
Hydrops testis	1	2	-
Mild ventriculomegaly	-	-	1
Visible bronchogram	-	-	1
Vacuolised placenta	-	1	-
**Total:**	**30**	**26**	**23**

## Data Availability

Not applicable.
